# Mechanisms and Novel Therapeutic Approaches for Gynecologic Cancer

**DOI:** 10.3390/biomedicines10051014

**Published:** 2022-04-28

**Authors:** Naomi Nakayama

**Affiliations:** Department of General Medicine and Community Health Science, School of Medicine, Hyogo Medical University, Hyogo 669-2321, Japan; na-nakayama@hyo-med.ac.jp; Tel.: +81-79-552-1181

The number of patients with gynecological cancers, such as ovarian and endometrial cancer, has been increasing worldwide. A possible cause of the high lethality of gynecological cancer is the lack of early detection tools and effective therapeutic interventions. In this regard, basic research on its pathophysiology and novel molecular-based therapeutic strategies is urgently required.

Recent research has focused on elucidating the tumor biology and molecular pathways that mediate cancer progression and drug resistance for the development of novel molecular-targeted therapies. These include monoclonal antibodies, small-molecule receptor tyrosine kinase inhibitors, and agents that block downstream signaling pathways in gynecological cancer. However, newly approved drugs for ovarian cancer are limited and the effectiveness of these incorporated therapies is limited. Therefore, further research is required to gain a better understanding of this phenomenon.

It was in this context that the Special Issue of *Biomedicines* entitled “Mechanisms and Novel Therapeutic Approaches for Gynecologic Cancer” was edited, focusing on how basic research, such as genomics, epigenomics, and proteomics, as well as clinical research can contribute to improving the mortality of patients with gynecological cancer.

This book, based on the aforementioned Special Issue of *Biomedicines*, contains a total of 13 papers (eight original research and five reviews) focusing on basic research of gynecologic cancer.

Among the original articles, the first study is an in vitro on ovarian cancer that focused on the novel ovarian cancer-related transcriptional factor, nucleus accumbens-associated protein 1 (NAC1). The researchers performed functional and structural analyses of its DNA-binding domain, the BEN domain, and clarified the target sequence using a PCR-assisted random oligonucleotide selection approach. The interaction between NAC1 and target DNA was validated using several novel techniques, including isothermal titration calorimetry (ITC), chromatin-immunoprecipitation assays, and NMR chemical shift perturbation (CSP). As NAC1 is significantly overexpressed in several types of carcinomas, including ovarian and cervical; is associated with tumor growth, survival and drug resistance; and is considered to be a target molecule for intervention, this study will contribute to novel molecular targeted therapies [1].

The second article describes a study of BRCA1 mutant ovarian cancer. They investigated the functional impact of platelet-activating factor acetylhydrolase (PAF-AH) and clarified its interaction with the Wnt signaling pathway. They then provided evidence that PAF-AH is a positive prognostic factor with functional impact mediated by the negative regulation of the Wnt/-catenin pathway in BRCA1 mutant ovarian cancer. This study shows the importance of PAF-AH as a biomarker for predicting disease risk in BRCA1 mutation carriers [2].

The third study incorporated gene ontology (GO)-based integrative approaches to explore the expression profiles of serous borderline ovarian tumors (BOTs) and serous ovarian carcinomas to identify common and meaningful dysregulated functions and dysfunctional pathways between these two groups. Then, they detected differentially expressed genes (DEGs), such as SRC, ARNT, TBP, and SNAI2, which play a crucial role in the pathogenesis of both tumors, implying a gradual evolution from serous BOTs to ovarian carcinomas. These findings may contribute to the future development of targeted therapies [3].

The fourth study describes clinical research on cervical cancer, in which HPV DNA tests are highly sensitive, but the specificity of HPV tests is low. This study identified the potential role of this test in diagnosing cervical precancerous lesions (CIN) using archival paraffin-embedded specimens of CIN1 (31), CIN2 (75), and CIN3. The authors concluded that, based on HPV-induced oncogenesis, the expression profile of testin, Ki-67, and p16 would improve test sensitivity and specificity in diagnosing cervical intraepithelial changes [4].

The fifth study was an in vitro study to establish an effective treatment strategy that targeted ovarian cancer stem cells (CSCs), which are related to chemoresistance and cancer recurrence. They developed a codon-optimized third-generation chimeric antigen receptor (CAR) to specifically target CD44, a CSC marker. They showed that simultaneous treatment with CD44NK and cisplatin demonstrated excellent antitumor activity against CD44+ ovarian cancer cells in vitro. This study provides the basis for further in vivo studies and future clinical development [5].

In the sixth study, the authors investigated somatic mutations in DNA damage response (DDR) genes in ovarian cancer tissue using a multi gene panel with next-generation sequencing. They discovered that DDR gene somatic mutations is more relevant in serous carcinoma and are associated with recurrence and cancer-related death. They then clarified the clinical characteristics and outcomes of ovarian cancer based on the DDR gene mutation profile. This study provides a rationale for future studies on novel therapeutic targets for DNA damage response pathways [6].

The seventh study describes clinical research on endometrioid endometrial cancer, showing the role of DNA mismatch repair (MMR) status in survival and its correlation with clinical prognostic factors in a relatively large sample size. The authors recruited 238 patients with endometrioid endometrial cancer and demonstrated that MMR deficiency (dMMR) is present in a significant number of patients and is associated with poorer clinicopathological factors and worse prognosis, particularly in long term follow-up (5–10 years). They concluded that dMMR should be considered in the risk stratification of endometrial cancer to guide optimal therapeutic intervention and individualization for a longer follow-up plan [7].

The last retrospective clinical research article focused on radiation therapy for endometrial cancer. The authors used in vivo dosimetry (IVD) to measure the dose to organs at risk (OAR) ratio for patients receiving postoperative high-dose-rate (HDR) interventional radiotherapy in two different fractionation schedules, analyzed its efficacy on treatment-related toxicities retrospectively, and showed its safety and acceptability [8].

Three review papers in the field of ovarian cancer were included in this book. First, there is a review on the human cytomegalovirus (HCMV) infection and ovarian cancer. The authors highlighted the impact of immunomodulatory effects of HCMV infection on host immune responses to ovarian carcinogenesis [9]. The second review focuses on ovarian cancer and exosomes, which play important roles in cell–cell communication and the regulation of various biological processes in cancer progression. The authors reviewed the potential of exosomal miRNAs in the circulation as a good biomarker for non-invasive early detection of ovarian cancer, along with current clinical trials [10]. In the third review of ovarian cancer, the authors comprehensively reviewed the molecular characteristics of ovarian cancer and the recent evidence on approved molecular targeted drugs, such as immune checkpoint inhibitors, PARP inhibitors, and anti-angiogenic therapies, which have made great advances in EOC treatment. They summarized new possible complementary approaches in preclinical stages, focusing on drug repurposing, non-coding RNAs, and nanomedicine as new methods for drug delivery [11].

The next review is on endometrial carcinoma (EC) and focuses on immune microenvironment modifications and immune response activation. The authors summarized the current knowledge of the immune environment of EC, both for mismatch repair deficient and mismatch repair proficient tumors. They also reviewed clinical data on immune checkpoint inhibitors (ICI) and PD-1/PD-L1 inhibitors and discussed the future possibility of various ICI-based combination therapies to limit resistance to immunotherapy [12].

The last review is a comprehensive literature review of vulvovaginal melanomas, which are quite rare among gynecologic cancers. Due to the lack of a sufficient number of cases to conduct randomized clinical trials, specific treatment guidelines have not yet been established, and the prognosis of vulvovaginal melanomas is very poor without a standardized treatment strategy. In this regard, this review is significant in highlighting the increasing research on the future establishment of novel therapeutic schemes [13].

I believe that this book includes important advanced studies on gynecologic cancer and comprehensive reviews covering relatively frequent to rare tumors in gynecologic oncology.
